# Characterization of the unique oral microbiome of children harboring *Helicobacter pylori* in the oral cavity

**DOI:** 10.1080/20002297.2024.2339158

**Published:** 2024-04-11

**Authors:** Yuko Ogaya, Tamami Kadota, Masakazu Hamada, Ryota Nomura, Kazuhiko Nakano

**Affiliations:** a Department of Pediatric Dentistry, Osaka University Graduate School of Dentistry, Osaka, Japan; b Department of Oral & Maxillofacial Oncology and Surgery, Osaka University Graduate School of Dentistry, Osaka, Japan; c Department of Pediatric Dentistry, Graduate School of Biomedical and Health Sciences, Hiroshima University, Hiroshima, Japan

**Keywords:** *Helicobacter pylori*, oral microbiome, oral cavity, saliva, metagenomic analysis

## Abstract

**Objective:**

*Helicobacter pylori* infection is acquired in childhood via the oral cavity, although its relationship with the characteristics of the oral microbiome has not been elucidated. In this study, we performed comprehensive analysis of the oral microbiome in children and adults with or without H. pylori in the oral cavity.

**Methods:**

Bacterial DNA was extracted from 41 adult and 21 child saliva specimens, and H. pylori was detected using PCR. 16S rRNA gene amplification was performed for next-generation sequencing. Bioinformatic analyses were conducted using Quantitative Insights into Microbial Ecology 2 (QIIME 2).

**Results:**

Faith’s phylogenetic diversity analysis showed a significant difference between H. pylori-negative adult and child specimens in terms of α-diversity (*p* < 0.05), while no significant difference was observed between H. pylori-positive adult and child specimens. There was also a significant difference in β-diversity between H. pylori-positive and negative child specimens (*p* < 0.05). Taxonomic analysis at the genus level revealed that Porphyromonas was the only bacterium that was significantly more abundant in both H. pylori-positive adults and children than in corresponding negative specimens (*p* < 0.01 and *p* < 0.05, respectively).

**Conclusion:**

These results suggest unique oral microbiome characteristics in children with H. pylori infection in the oral cavity.

## Introduction

*Helicobacter pylori* is a pathogenic bacterium involved in gastric diseases such as chronic gastritis, peptic ulcers, and gastric cancer [1]. It is estimated that the prevalence of *H. pylori* infection globally is approximately 50% [1,2], and its geographical variation is reasonably correlated with the incidence of gastric cancer [3]. In Japan, the majority of gastric cancer patients are infected with *H. pylori* [4]. *H. pylori* eradication is known to be effective at reducing the risk of gastric cancer [5], regardless of gastric symptoms such as peptic ulcer, dyspepsia, and abdominal pain [4]. A consensus report issued at the Taipei Global Consensus Meeting in 2020 suggested offering all *H. pylori*-infected individuals eradication treatment [6]. *H. pylori* eradication therapy requires 1 week of medication with three types of drugs, but a study has shown that drug-resistant *H. pylori* is gradually increasing in incidence [7]. Even after successful eradication, the recurrence of *H. pylori* infection is occasionally confirmed. The factors involved in the recurrence of *H. pylori* can be divided into *in vivo* factors, including oral colonization and transformation into a coccoid form, and *in vitro* factors, such as re-examination method and time, and treatment time window [8]. Numerous clinical studies have reported that gastric *H. pylori* infection is associated with oral *H. pylori* positivity [9,10], suggesting that the presence of *H. pylori* in the oral cavity is one of the most important causes of recurrence of *H. pylori* infection [8,11].

It is generally accepted that *H. pylori* can be transmitted through the oral cavity, such as via the oral – oral route, fecal – oral route, or gastro – oral route, during childhood [12]. Numerous studies have detected *H. pylori* in the oral cavity, including in the saliva [13,14], dental plaque [15], tongue coating [16], and dental pulp [17,18]. These findings suggest that this anatomical site is a reservoir for *H. pylori*. It has been reported that several oral bacteria interact with *H. pylori* in a way that may help it to survive in the oral cavity. For example, it has been reported that *H. pylori* can invade biofilm formed by *Streptococcus mutans* [19]. Meanwhile, *Porphyromonas gingivalis* and *Fusobacterium nucleatum* were found to coaggregate with *H. pylori*, while several oral bacteria were found to inhibit *H. pylori* [20]. However, these studies focused on specific, culturable bacteria.

More than 800 bacterial species inhabit the human oral cavity, including a large number of unculturable species [21]. Throughout childhood, the oral microorganisms increase in abundance; the initial colonizers are related to various factors, such as personal relationships and the living environment [22]. These early oral microbial communities play a major role in the development of the adult oral microbiome, and are potentially a source of both pathogenic and protective microorganisms [22]. Hence, as the oral cavity is a gateway for *H. pylori* infection, analysis of the characteristics of the oral microbiome in the presence of *H. pylori* would be meaningful to deepen our understanding of *H. pylori* infection. The possibility of *H. pylori* infection altering the oral microbiome has been reported [23,24], but no report discussing this possibility in children has been published.

In the present study, we investigated and compared the microbiomes in the oral cavity of *H. pylori*-positive or -negative children and adults using next-generation sequencing to examine bacteriological factors that could be related to *H. pylori* infection.

## Materials and methods

### Ethics statement

This study was conducted in compliance with the Declaration of Helsinki. This study was approved by the Osaka University Graduate School of Dentistry ethics committee (no. H30-E-32). All study participants and their guardians were informed of the study contents, and written informed consent was obtained for all participants before specimen collection.

### Subjects and collection of oral specimens

This study included 41 adults (age range 20–74 years) who were referred to the Department of Oral and Maxillofacial Surgery at Osaka University Dental Hospital from July 2019 to January 2021 because of problems with a third molar that necessitated its extraction. This study also included 21 children and adolescents (age range 1–12 years) who attended Osaka University Dental Hospital from October 2017 to February 2018 for treatment or a regular checkup (Table 1). Dental plaque and dental pulp specimens from adults were collected from extracted teeth. Meanwhile, dental plaque was collected from children using sterilized instruments from the anterior and posterior teeth on both sides of the maxilla and mandibular jaw, and root canal specimens were collected using sterile root canal instruments from those who underwent root canal treatment. Both specimens were stored in 1 mL of sterile saline in sterile plastic tubes. Saliva from the adults and the children was collected in a sterile plastic tube. Bacterial DNA was extracted from these saliva, dental plaque, and dental pulp specimens as described below and used to determine the presence of oral *H. pylori*.

**Table 1.** Number of study subjects.

Clinical characteristics	Adults (mean age ± SD)	Children (mean age ± SD)
Total number	41 (37.12 ± 16.46)	21 (8.00 ± 2.39)
Sex		
Male	15 (35.47 ± 16.21)	13 (7.85 ± 2.41)
Female	26 (38.08 ± 16.53)	8 (8.25 ± 2.33)
*H. pylori* detection in oral cavity		
Detection rate (%)	46.3	38.1

### H. pylori strains and growth conditions

*H. pylori* reference strain J99 (ATCC 700824) was purchased from Summit Pharmaceuticals International Corporation (Tokyo, Japan) and served as a positive control for PCR assay. The strain was cultured on blood agar plates (Becton Dickinson, Franklin Lakes, NJ, USA) and incubated at 37°C for 3–5 days to isolate bacterial colonies [18]. Thereafter, colonies were inoculated into 10 mL of Brucella Broth (Becton Dickinson) supplemented with 1 mL of horse serum using a sterilized platinum loop and incubated at 37°C for 24 h under microaerophilic conditions. Centrifugation was performed at 8,000 rpm for 10 min to collect the bacteria, and genomic DNA was extracted as described below.

### Bacterial DNA extraction

Using a previously reported method, bacterial DNA was extracted from oral specimens and *H. pylori* strain J99 [18]. Briefly, oral specimens or *H. pylori* strain J99 were resuspended in 250 µL of 10 mM Tris-HCL (pH 8.0) containing 100 mM NaCl and 1 mM EDTA. The cells were collected by centrifugation and lysed in 600 µL of Cell Lysis Solution (Qiagen, Düsseldorf, Germany) for 5 min at 80°C. The cell lysate was incubated with 3 µL of RNase (10 mg/mL; Qiagen) for 30 min at 37°C followed by the addition of Protein Precipitation Solution (Qiagen), vigorously vortexed for 20 s, and centrifuged at 10,000 × *g* for 3 min. Genomic DNA was extracted by adding 600 µL of 2-propanol (Wako Pure Chemical Industries, Tokyo, Japan) to the supernatant and collected by centrifugation. The DNA pellet was washed with 70% ethanol (Wako Pure Chemical Industries), centrifuged, air-dried, and dissolved in 100 µL of Tris-EDTA buffer [10 mM Tris-HCL, 1 mM EDTA (pH 8.0)].

### H. pylori PCR detection

To detect *H. pylori* in the oral cavity, nested PCR was performed with bacterial DNA extracted from all oral specimens using *H. pylori*-specific primer sets described in our previous report [17]. The nested PCR consisted of two-step PCR assays. Briefly, a first-step PCR assay was performed using 2 µL of bacterial DNA extracted from oral cavity specimens and amplified in a 20 µL reaction mixture containing the primers *ureA*-aF and *ureA*-bR. For the second-step PCR, 1 µL of the first PCR product was used as a template for a 20 µL reaction mixture containing the primers *ureA*-bF and *ureA*-aR. The cycles of both first- and second-step PCR were performed via a single operation using TAKARA Ex *Taq* polymerase (Takara Bio Inc., Otsu, Japan), as described previously [18]. The PCR products were fractionated using 1.5% (*w/v*) agarose gel containing Tris-acetate-EDTA buffer, stained with ethidium bromide (0.5 µg/mL), and visualized under UV illumination.

### 16S rRNA gene library preparation, sequencing, and operational taxonomic units (OUT) analysis

To analyze the oral microbiome, bacterial DNA from saliva specimens was used for 16S rRNA gene sequencing. Bioinformatic analyses were conducted using Quantitative Insights into Microbial Ecology 2 (Qiime 2) [25]. The quality of the reads obtained from all specimens was evaluated with Qiime2, and only reads considered to be of sufficient quality were included in the subsequent analysis. An OTU table was made using Dada2. Thereafter, diversity analysis and taxonomic classification were performed.

### Statistical analysis

Faith’s phylogenetic diversity analysis was performed to compare *H. pylori*-positive and negative specimens from adults and children, and Mann – Whitney *U* test was performed to analyze the differences between each group. β-Diversity analysis was performed to compare *H. pylori*-positive and -negative specimens from adults and children using weighted UniFrac distance, and the results were visualized on a principal coordinate analysis (PCoA) plot. Then, permutational multivariate analysis of variance (PERMANOVA) was performed to evaluate the statistical significance of differences between groups. Phyla represented by taxa with mean relative abundance in excess of 1% were extracted, as were genera for which OTUs were present in more than 50% of the specimens. The differences in relative abundances of the constituent bacteria between each group were analyzed using Mann – Whitney *U* test. In all analyses, a significance level of *p* < 0.05 was considered to be significant.

## Results

### Study subjects and H. pylori detection

A total of 41 adults (15 men and 26 women; mean age, 37.12 ± 16.46; age range 20–74 years) and 21 children (13 boys and 8 girls; mean age, 8.00 ± 2.39; age range 1–12 years) were enrolled in this study (Table 1). *H. pylori* was detected from oral specimens collected from all subjects using nested PCR with previously described *H. pylori*-specific primer sets [17]. *H. pylori* was detected from 46.3% of adults (9 men and 10 women; age range 20–70) and 38.1% of children (6 boys and 2 girls; age range 4–10) (Table 1).

### α-diversity analysis

**Figure 1.**
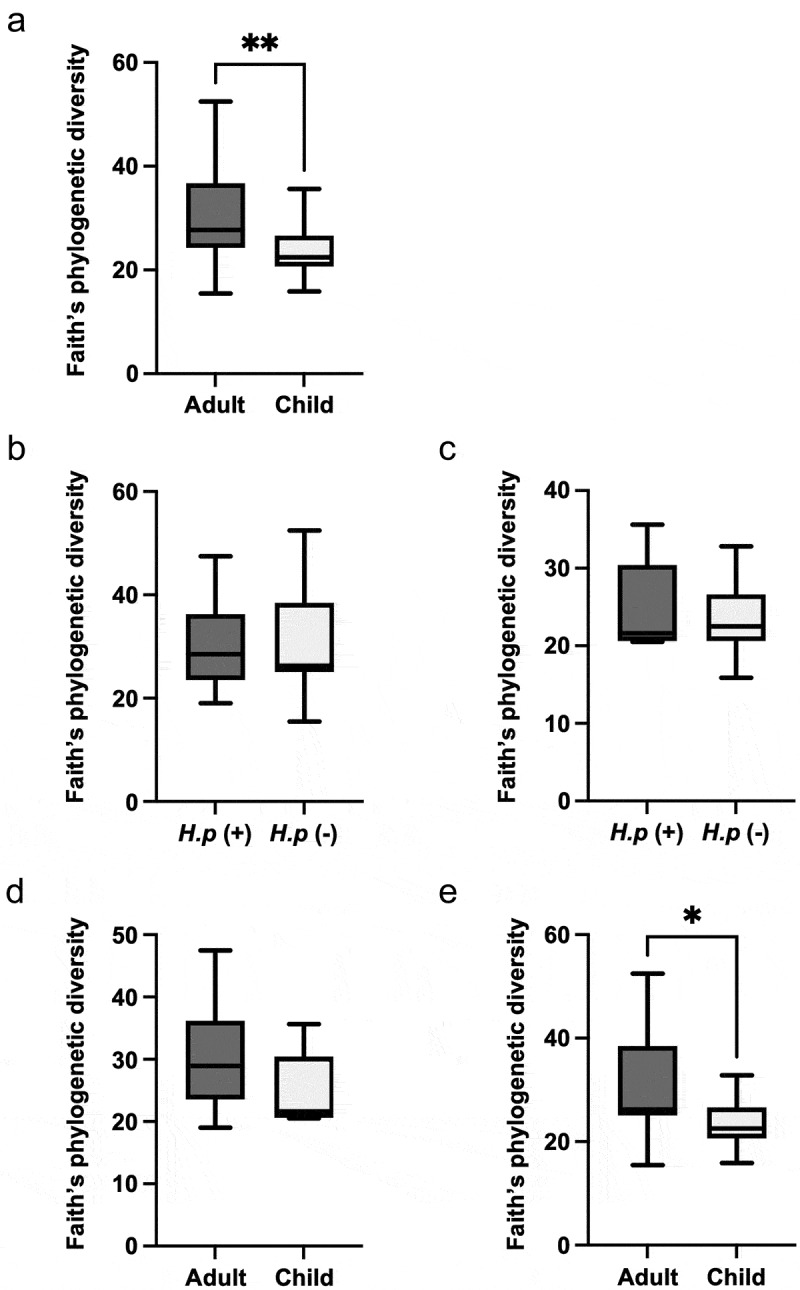
Faith’s phylogenetic diversity analysis of α-diversity. (a) Comparison between adults and children in all specimens. Comparisons between (b) *H. pylori*-positive and -negative specimens from adults, and (c) *H. pylori*-positive and -negative specimens from children. Comparisons between (d) *H. pylori*-positive adults and children, and (e) *H. pylori*-negative adults and children. Whiskers indicate maximum and minimum values, while boxes indicate interquartile ranges. *P<0.05 and **P<0.01 by Mann–Whitney *U* test.

Next-generation sequencing of the V3–V4 hypervariable region of bacterial 16S rRNA extracted from saliva specimens of adults and children was performed, followed by bioinformatic analyses. Faith’s phylogenetic diversity analysis revealed that α-diversity in the adult specimens was significantly greater than that in the child specimens (*p* < 0.001) (Figure 1(a)). No significant difference in α-diversity was shown between *H. pylori*-positive and -negative adult specimens or between *H. pylori*-positive and -negative child specimens (Figure 1(b,c)). Furthermore, no significant difference was observed between *H. pylori*-positive adult and *H. pylori*-positive child specimens (Figure 1(d)). Meanwhile, α-diversity of *H. pylori*-negative specimens from adults was significantly greater than that of *H. pylori*-negative specimens from children (*p* < 0.05) (Figure 1(e)).

### β-diversity analysis

**Figure 2.**
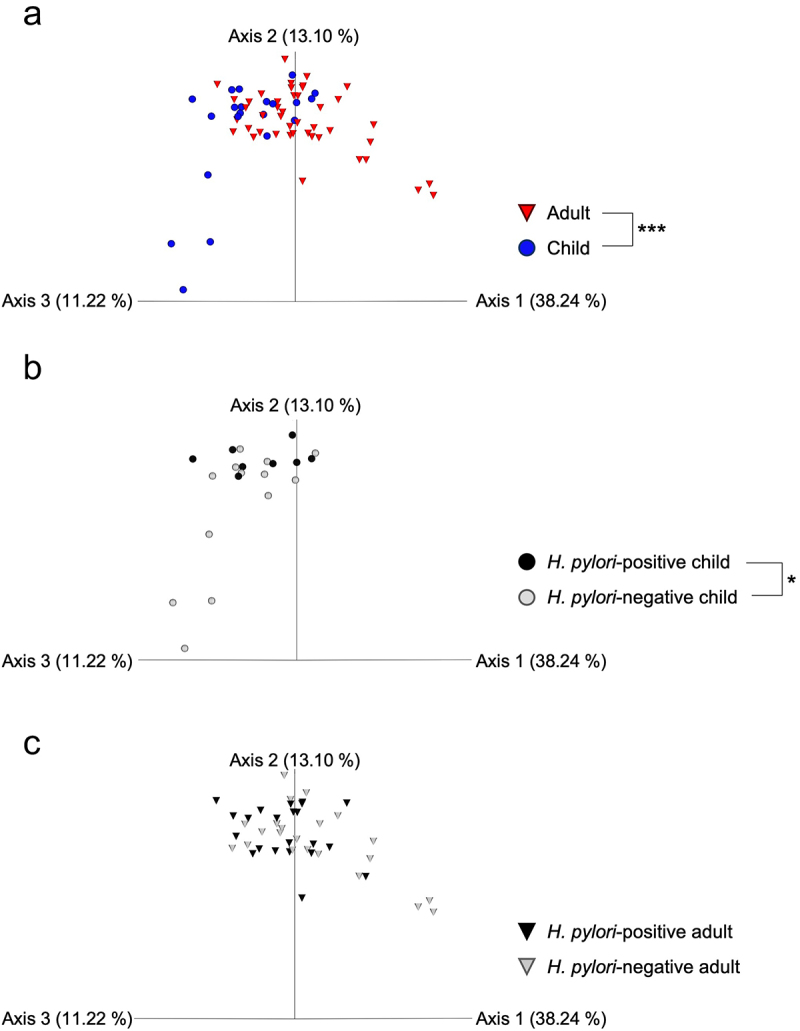
PERMANOVA of β-diversity in *H. pylori*-positive and -negative groups of adults and children. (a) Comparison between adult *H. pylori*-negative specimens and child *H. pylori*-positive/-negative specimens. (b) Comparison between adult *H. pylori*-positive specimens and child *H. pylori*-positive/-negative specimens. (c) Comparison between child *H. pylori*-positive and -negative specimens. The contribution rate of each axis (1–3) in the principal coordinate analysis (PCoA) is shown in parentheses. *p < 0.05 by PERMANOVA of the weighted UniFrac distance.

β-Diversity was analyzed using PCoA of the weighted UniFrac distance, and oral microflora was compared between *H. pylori*-positive and -negative cases and between adults and children. There were significant differences between adult specimens and child specimens (*p* < 0.001) (Figure 2(a)). *H. pylori*-positive and -negative specimens from children also showed significant differences in β-diversity (*p* < 0.05) (Figure 2(b), while no significant differences were observed between *H. pylori*-positive and -negative specimens from adults (Figure 2(c)).

### Taxonomic analysis at the phylum level

**Figure 3.**
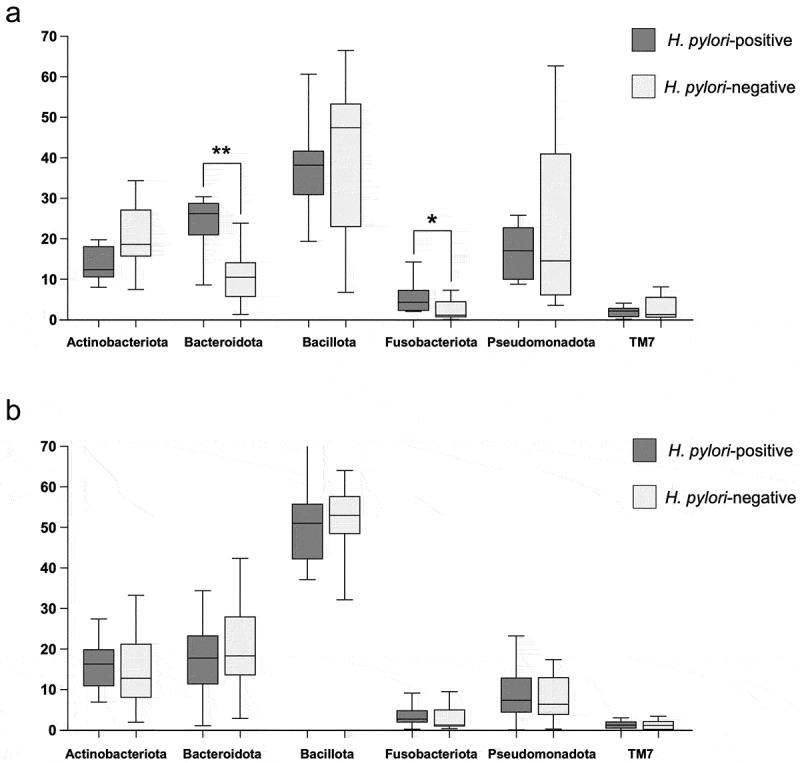
Taxonomic analysis at the phylum level in *H. pylori*-positive and *H. pylori-*negative specimens. (a) Children. (b) Adults. Whiskers indicate maximum and minimum values, while boxes indicate interquartile ranges. *p < 0.05 and **p < 0.01 by Mann–Whitney *U* test.

Taxonomic analysis revealed 16 phyla among all specimens. Six had a relative abundance of approximately 2% or more and accounted for over 96% of the total. Among them, Bacteroidota and Fusobacteriota showed higher relative abundance in *H. pylori*-positive specimens than in *H. pylori*-negative specimens from children (*p* < 0.01 and *p* < 0.05 respectively) (Figure 3(a)). In contrast, no significant differences were observed between *H. pylori*-positive and -negative specimens from adults (Figure 3(b)).

### Taxonomic analysis at the genus level

**Figure 4.**
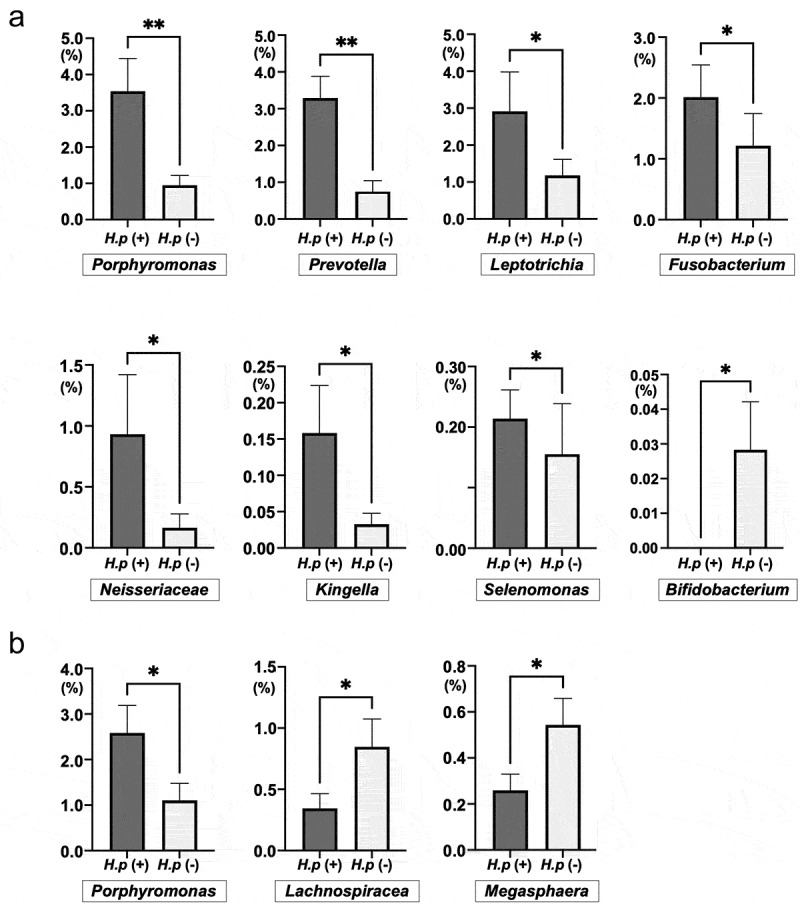
Taxonomic analysis at the genus level in *H. pylori*-positive and *H. pylori-*negative specimens. (a) Children. (b) Adults. *p < 0.05 and **p < 0.01 by Mann–Whitney *U* test.

Among all the specimens, a total of 258 genera were observed. Overall, 248 of these were detected in adults, which was significantly more than the 116 genera detected in children (*p* < 0.001). Eight bacterial genera showed significant differences in relative abundance between *H. pylori*-positive and -negative specimens from children. Among these genera, *Porphyromonas*, *Fusobacterium*, *Leptotrichia*, *Selenomonas*, *Prevotella*, *Neisseriaceae*, and *Kingella* showed significantly higher relative abundance in *H. pylori*-positive specimens than in negative ones (*p* < 0.05) (Figure 4(a)). Only *Bifidobacterium* was more abundant in *H. pylori*-negative specimens (*p* < 0.05). *Porphyromonas*, *Lachnospiraceae*, and *Megasphaera* showed significant differences in relative abundance between *H. pylori*-positive and -negative specimens (*p* < 0.05) (Figure 4(b)). Only *Porphyromonas* showed greater relative abundance in both child and adult *H. pylori*-positive specimens than in negative ones.

## Discussion

In the present study, we performed a metagenomic analysis of the oral bacteria in the presence and absence of *H. pylori* in adults and children to investigate the characteristics of the oral microbiome that may be specific to *H. pylori* infection in the oral cavity.

Here, oral *H. pylori* infection was observed in 46.3% of adults and 38.1% of children. The rate of oral *H. pylori* infection was previously reported to be approximately 25% in adults and about 40% in children [17,26]. The estimated global prevalence of *H. pylori* has decreased globally over the last 40 years, dropping from 58.2% in the period of 1980–90 to 43.1% in 2011–2022 [27]. This trend has been mirrored in each region worldwide: 85.1% in 1980–90 to 53.3% in 2011–22 in Africa, 59.2% to 44.2% in Europe, and 56.5% to 32.8% in the Americas [27]. In Japan, the prevalence of *H. pylori* is approximately 8%–60% for those aged 20 to 74 years [28] and approximately 0%–5% for those under 12 years old [29]. This latter rate is particularly low compared with the rate of *H. pylori* in the oral cavity in a similar age group as reported in this study. *H. pylori* is believed to be transmitted through infected food and water or via the oral route from the mother [30], and the oral cavity can serve as a reservoir, either temporarily or persistently. This could explain why higher detection rates were observed, especially in young individuals, compared with the rate of *H. pylori* infection in the stomach.

Although 16S rRNA sequences can be used to identify species to some extent, they were not used to detect *H. pylori* in the oral cavity here because a previous study suggested the unsuitability of such an approach [18]. Therefore, *H. pylori*-specific primer sets were instead applied to detect *H. pylori* in this study. To evaluate *H. pylori* infection here, the oral cavity was judged to be positive for this species if it was detected in any of the dental plaque, dental pulp, or saliva specimens collected from the oral cavity. The oral cavity contains a variety of bacterial flora, which varies from site to site. By analyzing multiple oral specimens, we were able to more accurately examine oral cavities that were definitively *H. pylori*-positive. Since saliva includes the microorganisms shed from the flora at various oral sites, it is considered to serve as a fingerprint for the entire oral microbiota [31]. Therefore, to examine the flora of the whole oral cavity, a metagenomic analysis was performed on saliva specimens. Furthermore, as *H. pylori* transits through saliva, it would be meaningful to detect the effect of its infection on the salivary microbiome. However, a recent study confirmed that the microbiota in oral rinse fluid specimens is more representative of the entire site-specific microbiota than that found in saliva [32]. Consequently, the analysis of rinse fluid specimens rather than saliva specimens could be considered.

Faith’s phylogenetic diversity analysis revealed significant differences between adult and child oral microbiomes. Significant differences of this kind were also identified between *H. pylori*-negative adults and children. Significant differences were also identified between adult and child microbiomes in β-diversity analysis. These results suggest that adults exhibit greater phylogenetic richness in bacterial species than children. The oral microbial community gradually matures and tends to stabilize with growth and development [33]. During and after birth, newborns are exposed to a variety of microorganisms, but only some of them colonize the oral cavity [34]. Upon eruption of the primary teeth, the bacterial diversity and richness in the oral cavity increase. It was reported that the saliva microbiome of primary dentition demonstrated greater alpha diversity than that of predentate infants [35]. Given that the child participants in this study were between 4 and 12 years of age, at which stage the oral microflora is considered to still be immature compared with that of adults, the results are consistent with these reports. Meanwhile, no significant differences were observed between *H. pylori*-positive and -negative oral microbiomes in both adults and children in Faith’s phylogenetic diversity analysis, indicating that phylogenetic richness does not differ regardless of the existence of *H. pylori*. In contrast to the results of α-diversity, there were significant differences in β-diversity between *H. pylori*-positive and -negative specimens from children. As β-diversity assesses the diversity of microbial communities across different specimens, the findings suggest that diversity among *H. pylori-*positive specimens is significantly higher than that among *H. pylori*-negative specimens.

In the analysis at the phylum level, 6 of the 16 phyla with a relative abundance of 2% or higher were most dominant in all groups. Of these six phyla, Bacteroidota and Fusobacteriota were significantly more abundant in *H. pylori*-positive specimens from children than in negative ones. Some studies that examined the oral microbiota in adults indicated that Bacteroidota was more abundant in *H. pylori*-positive specimens than in negative ones [16,36]. In this study, similar results were obtained from the child oral microbiome.

The findings on relative abundance at the genus level revealed that *Porphyromonas* was the only genus that exhibited higher abundance in *H. pylori*-positive specimens than in negative ones in both adults and children. *Porphyromonas* is a gram-negative, obligately anaerobic genus characterized by the production of porphyrin pigments [37]. The most well-known species of this genus is *Porphyromonas gingivalis*, which has long been studied as an important pathogen associated with human periodontal disease [38]. Numerous clinical studies have demonstrated the correlation between the presence of *H. pylori* and periodontitis [39–41]. Some reports indicated that *H. pylori* is frequently detected in dental plaque specimens from deep periodontal pockets, particularly with the presence of a specific genotype of *P. gingivalis* [26]. Recently, direct interaction between *P. gingivalis* and *H. pylori* was demonstrated, with pre-incubation of *P. gingivalis* with *H. pylori* being shown to enhance *P. gingivalis* biofilm formation and facilitate bacterial internalization into oral keratinocytes [42]. Another well-known species in this genus, *Porphyromonas endodontalis*, is primarily found in infections that originate in the pulp [43,44], but it has also been reported to have a high prevalence in sites affected by periodontitis, similar to *P. gingivalis*, without periapical lesions [45]. This suggested the possibility of there being a strong association between *Porphyromonas* and *H. pylori*, which is considered to support our findings.

Overall, there were few differences in the oral microbiome between *H. pylori*-positive and -negative adult specimens, compared with the corresponding findings on the oral microbiome from child specimens. This could be attributed to the use of a highly sensitive nested PCR method for detecting *H. pylori* [17], suggesting that there was not sufficient *H. pylori* to induce significant changes in the mature oral microbiome of adults.

To the best of our knowledge, no studies have investigated the oral microbiome of children specifically focusing on oral *H. pylori*, and this study is the first to report on it. In this study, we were able to identify differences in the oral microbiome based on the presence or absence of *H. pylori*. However, this study is limited by its small sample size. Therefore, further studies with larger sample sizes should be conducted to confirm the validity of our results for all oral *H. pylori*-positive/-negative children.

## Conclusions

In summary, the oral microbiome of *H. pylori*-positive children was more diverse than that of *H. pylori*-negative children. In both adults and children, *Porphyromonas* was more abundant in the *H. pylori*-positive oral microbiome than in the *H. pylori*-negative oral microbiome. These results suggest unique characteristics of the oral microbiome in those with *H. pylori* infection in the oral cavity, particularly in children.
